# Differentiation of Human Induced Pluripotent Stem Cells from Patients with Severe COPD into Functional Airway Epithelium

**DOI:** 10.3390/cells11152422

**Published:** 2022-08-05

**Authors:** Engi Ahmed, Mathieu Fieldes, Chloé Bourguignon, Joffrey Mianné, Aurélie Petit, Myriam Jory, Chantal Cazevieille, Hassan Boukhaddaoui, James P. Garnett, Christophe Hirtz, Gladys Massiera, Isabelle Vachier, Said Assou, Arnaud Bourdin, John De Vos

**Affiliations:** 1 IRMB, University Montpellier, INSERM, CHU Montpellier, F-34000 Montpellier, France; 2 Department of Respiratory Diseases, University Montpellier, CHU Montpellier, Hôpital Arnaud de Villeneuve, F-34000 Montpellier, France; 3 Centre National de la Recherche Scientifique UMR 5221, Laboratoire Charles Coulomb, University Montpellier, F-34000 Montpellier, France; 4 Institut des Neurosciences de Montpellier, University Montpellier, F-34000 Montpellier, France; 5 Montpellier Ressources Imagerie (MRI), University Montpellier, F-34000 Montpellier, France; 6 Immunology & Respiratory Diseases Research, Boehringer Ingelheim Pharma GmbH & Co. KG, 55216 Biberach an der Riss, Germany; 7 Translational and Clinical Research Institute, Newcastle University, Newcastle upon Tyne NE2 4HH, UK; 8 Centre PhyMedExp, University Montpellier, INSERM U1046, F-34000 Montpellier, France; 9 Department of Cell and Tissue Engineering, University Montpellier, CHU Montpellier, F-34000 Montpellier, France; 10 Hôpital Saint-Eloi, 80 Avenue Augustin Fliche, CEDEX 5, F-34295 Montpellier, France

**Keywords:** airway epithelium, chronic obstructive pulmonary disease, disease modeling, human induced pluripotent stem cells

## Abstract

Background: Chronic Obstructive Pulmonary Disease (COPD), a major cause of mortality and disability, is a complex disease with heterogeneous and ill-understood biological mechanisms. Human induced pluripotent stem cells (hiPSCs) are a promising tool to model human disease, including the impact of genetic susceptibility. Methods: We developed a simple and reliable method for reprogramming peripheral blood mononuclear cells into hiPSCs and to differentiate them into air–liquid interface bronchial epithelium within 45 days. Importantly, this method does not involve any cell sorting step. We reprogrammed blood cells from one healthy control and three patients with very severe COPD. Results: The mean cell purity at the definitive endoderm and ventral anterior foregut endoderm (vAFE) stages was >80%, assessed by quantifying C-X-C Motif Chemokine Receptor 4/SRY-Box Transcription Factor 17 (CXCR4/SOX17) and NK2 Homeobox 1 (NKX2.1) expression, respectively. vAFE cells from all four hiPSC lines differentiated into bronchial epithelium in air–liquid interface conditions, with large zones covered by beating ciliated, basal, goblets, club cells and neuroendocrine cells, as found in vivo. The hiPSC-derived airway epithelium (iALI) from patients with very severe COPD and from the healthy control were undistinguishable. Conclusions: iALI bronchial epithelium is ready for better understanding lung disease pathogenesis and accelerating drug discovery.

## 1. Introduction

Chronic obstructive pulmonary disease (COPD) is a chronic lung disease characterized by respiratory symptoms associated with chronic airflow limitation. COPD is the third leading cause of death worldwide, and affects approximately 300 million people in the world [1]. Although cigarette smoking has been considered the most frequent cause of COPD, about half of cases are linked to non-tobacco-related risk factors, such as outdoor air pollution, biomass smoke, and occupational exposure to fumes and dust [2]. In COPD, the small conducting airways (<2 mm in diameter) are the major site of airflow obstruction, inflammation, and destruction [3,4,5]. Therefore, in vitro bronchial epithelium models are crucial to better understand and treat COPD.

Induced pluripotent stem cells (iPSCs) represent an attractive solution to model chronic airway diseases because they can yield a virtually unlimited amount of any differentiated cell type [6]. Recently described protocols to differentiate human pluripotent stem cells (PSCs) into bronchial epithelium [7,8,9,10,11,12,13,14,15] rely on the knowledge gathered on normal lung development in mammals [16]. Briefly, lung embryogenesis starts with the definitive endoderm (DE) formation. During the 4th week of human embryonic development, the primitive gut appears and can be divided into foregut, midgut, and hindgut. Early pulmonary development starts from the ventral area of the anterior foregut endoderm (vAFE). From this zone, which is characterized by the expression of the transcription factor NK2 homeobox 1 (NKX2.1), the respiratory diverticulum emerges and forms the trachea, and then bronchi, bronchioles, and alveoli. These steps can be recapitulated in vitro by differentiating PSCs first into DE and then into vAFE [17]. Finally, vAFE cells are differentiated into lung progenitors and bronchial cells. However, the protocols for PSC differentiation into bronchial epithelium present several drawbacks, and many of them have been rarely described in detail. In addition, many of these protocols work only with some pluripotent stem cell lines, often cell lines derived from healthy controls, and require an enrichment step based on the specific selection of NKX2.1+ cells at the vAFE stage using flow cytometry and cell surface markers (e.g., carboxypeptidase M+ cells [13] or CD47^high^ CD26^low^ cells [18]), or a final differentiation step in 3D culture conditions. Others require important technical skills and are difficult to replicate [19].

Here, we developed an easy approach to differentiate human iPSCs (hiPSCs) into proximal airway epithelium, without any cell purification steps. Careful in-home reprogramming and then culture adaptation to single-cell passaging, together with precise timing and reagent benchmarking for each differentiation step, led to the successful generation of fully differentiated and functional bronchial epithelium in air–liquid interface (ALI) culture conditions from four hiPSC lines (iALI bronchial epithelium), among which, three were derived from patients with severe COPD. This study highlights the crucial importance of evaluating the cell expansion and differentiation conditions for achieving optimal phenotypic and functional endpoints, such as ciliary beat frequency (CBF), mucus flow velocity, differentiated cells, and transepithelial electrical resistance (TEER). This simple protocol to produce hiPSC-derived iALI bronchial epithelium will facilitate airway disease modeling for developing novel gene/cell therapies, and for drug discovery. 

## 2. Materials and Methods

### 2.1. Patients’ Clinical Characteristics

Patients were younger than 55 years and had severe, early onset COPD (i.e., ratio of forced expiratory volume in one second (FEV1) to forced vital capacity (FVC) <0.70 and FEV1% predicted <50% on postbronchodilator spirometry). More clinical data are available in Supplementary Figure S1 and Appendix A.

### 2.2. Human Embryonic Stem Cell (ESC) and hiPSC Generation and Maintenance

The hiPSC lines HY03 (UHOMi002-A) (healthy control), iCOPD2 (UHOMi003-A), iCOPD8 (UHOMi004-A), and iCOPD9 (UHOMi005-A) were reprogrammed from peripheral blood mononucleated cells (PBMCs) using the StemSpan with Erythroid Expansion Medium (SSEM, StemCell Technologies, Vancouver, BC, Canada) and the CytoTune^®^-iPS 2.0 Sendai Reprogramming Kit (Thermo Fisher Scientific, Waltham, MA, USA, cat.no A16517), according to the manufacturer’s instructions [20,21]. Emerging hiPSC clones were mechanically selected and clonally expanded using mechanical passaging at early (<10) passages. At least three clones for each donor were maintained and their genetic stability was confirmed (Supplementary Figure S2). Pluripotency was confirmed by alkaline phosphatase activity staining, stage-specific embryonic antigen 3/4 (SSEA3/4), and TRA1-60 cell surface expression by flow cytometry, as previously published [22]. The human ESC line HD291 was derived in our laboratory [23]. PSC lines were maintained in an undifferentiated state in feeder-free conditions on growth factor-reduced Geltrex (Thermo Fisher Scientific) in E8 medium (Thermo Fisher Scientific). Cells were cultured in 35 mm dishes at 37 °C and were dissociated mechanically (under an optical microscope) or into single cells at 90% of confluence (every 4–5 days). Single-cell passaging was performed by adding Versene (Thermo Fisher) at 37 °C for 5 min and then seeding at 1:10–1:20 ratio with 10µM Y-27632 (Tocris), a potent and selective inhibitor of Rho-associated coiled-coil containing protein kinase (ROCK). The E8 maintenance medium was changed every day.

### 2.3. HIPSC Differentiation

**Figure 1:**
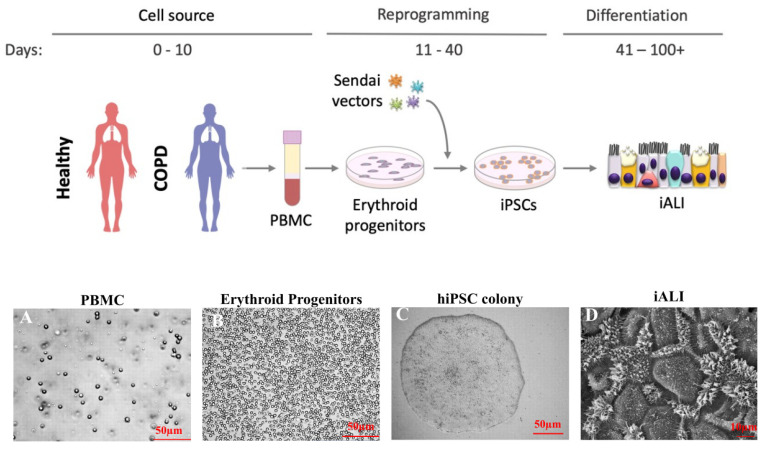
Study protocol: from human induced pluripotent stem cell (hiPSC) generation to hiPSC-derived airway epithelium. Recovery of cell source (day 0 to day 10). Peripheral blood mononuclear cells (PBMC) are isolated from whole blood samples from healthy controls (HY03) and patients with chronic obstructive pulmonary disease (COPD) (COPD 2, 8 and 9). (**A**) Optical microscopy image of PBMCS, scale bar: 50 µm). Then, the CD34+ subpopulation is amplified into erythroid progenitor cells (**B**) optical microscopy image, scale bar: 50 µm). Cell reprogramming (day 11 to day 40). Erythroid progenitors are transduced using Sendai virus-based constructs to express OCT3/4, SOX2, KLF4, and c-Myc. HiPSC colonies are visible at day 40 (**C**) optical microscopy image, scale bar: 50 µm). HiPSC differentiation into airway epithelium (iALI; day 41 to >day 100) (**D**) electronic microscopy image of iALI from the control HY03 hiPSC line, scale bar: 10 µm).

**Figure 2:**
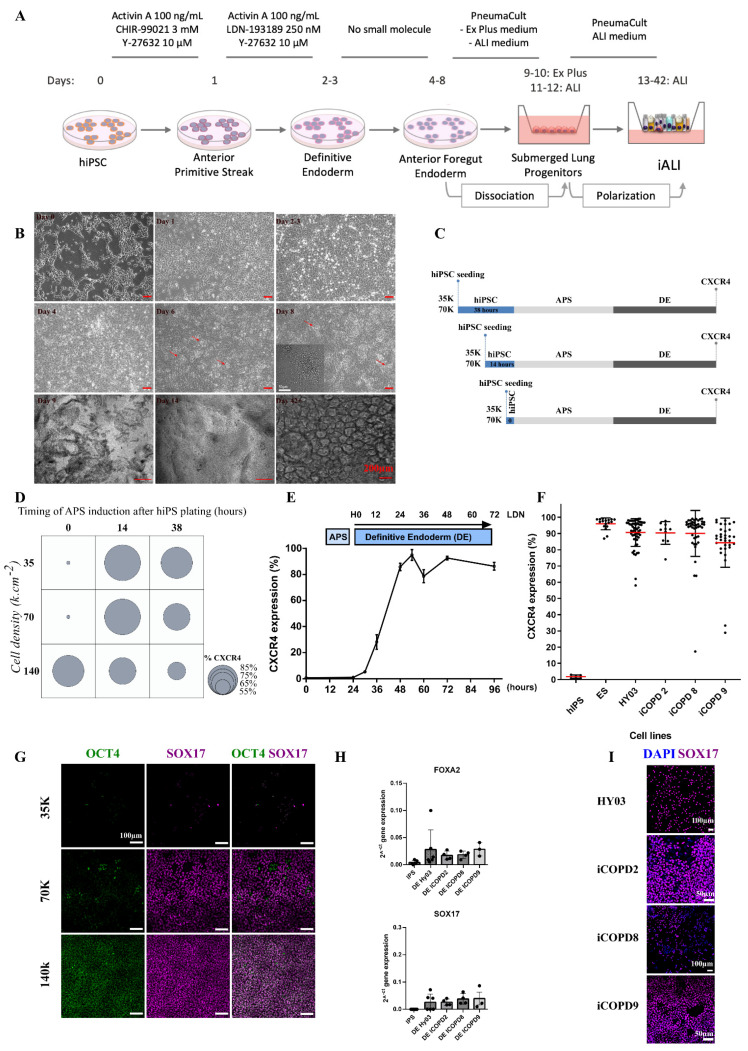
Differentiation of human induced pluripotent stem cells (hiPSC) into bronchial airway epithelium. (**A**) Schematic representation of the differentiation protocol. (**B**) Morphological changes during the various differentiation steps. Day 0: hiPSCs plated as single cells. Day 1: anterior primitive streak (APS). Day 2–3: definitive endoderm (DE). Day 4, 6, and 8: anterior foregut endoderm; red arrows: bud-like structures. Day 9: lung progenitors after mechanical clump passage and plating on Transwell inserts. Day 14 (polarization day): epithelial layer. Day 42+: multi-ciliated bronchial epithelial layer. Scale bar: 200 µm. (**C**) Experiment to optimize the interval between hiPSC plating and APS induction (two plating densities: 35,000 and 70,000 cells/cm^2^). (**D**) Results of the optimization experiment based on the percentage of CXCR4-expressing cells, assessed by flow cytometry (DE marker). Low cell density plating and short interval before APS induction led to hiPSC death. A long interval before hiPSC differentiation induction led to non-optimal DE induction. On the basis of these results, the density of 70 K·cm^2^ and APS induction at 14 h after hiPSC plating were selected as optimal conditions. (**E**) Time course of DE induction (n = 3, HY03 hiPSC line); similar results were obtained for the other cell lines. Maximum CXCR4 expression was reached at 24–48 h after DE induction. (**F**) Quality of DE induction based on CXCR4 expression by flow cytometry analysis in the five different PSC lines used (at least n = 8 independent experiments for each cell line). hiPS: undifferentiated hiPSC (negative control). (**G**) Immunolabeling at the DE stage for SOX17 (endoderm) and OCT4 (pluripotency marker). Too low (35 K·cm^−2^) and too high (140 K·cm^−2^) cell density at plating led to massive cell loss and incomplete OCT4 inhibition, respectively. Optimal cell density (here, 70 K·cm^−2^) induced strong OCT4 inhibition and high SOX17 expression. Images are for the iCODP8 cell line, but similar results were obtained also for the control HY03 iPSC line. Scale bar: 100 µm. (**H**) Expression of DE-specific genes (*SOX17* and *FOXA2*) at the DE stage (day 2–3 of differentiation). (**I**) Immunolabeling for SOX17 (DE marker) at day 2–3 of differentiation for each cell line. Scale bar: 100 µm (HY03 cells and iCOPD8); 50 µm (iCOPD2 and 9 cells).

Differentiation was carried out as described in Figure 1 and Figure 2A, using reagents at the concentrations listed in Supplementary Tables S1 and S2. Cells were plated at high-density (one 35 mm dish for two Transwell inserts) on Geltrex-coated Transwell inserts. During differentiation in hypoxic conditions (5% O_2_, 37 °C), medium was changed every day. 

### 2.4. Statistical Analysis

Data are presented as mean and standard deviation (SD) or standard error of the mean (SEM), and graphs were generated with GraphPad (GraphPad Software Prism, v 6.01, San Diego, CA, USA). All shown data are from experiments repeated at least three time. *p* < 0.05 indicated significant differences between groups.

Ethical approval: INVECCO study (INVECCO ClinicalTrials.gov Identifier: NCT03181204, CPP Sud Med IV) under the number ID RCB: 2017-A00252-51, CPP reference: protocol 08-2017, Promoter University Hospital of Montpellier. 

## 3. Results

### 3.1. Reprogramming from Blood Samples

We reprogrammed PBMCs from one healthy control and the three patients with severe COPD to generate the HY03, iCOPD2, iCOPD8, and iCOPD9 hiPSC lines, respectively (Figure 1) [20,21]. We isolated PBMCs with Ficoll (Figure 1A) and cultured them using the STEM SPAN SFEMII^®^ Kit enriched with cytokines (interleukin 3, stem cell factor, erythropoietin) to promote Erythroid Progenitor (EP) expansion (Figure 1B). We monitored CD45, CD34, CD71, and CD36 expression to optimize EP expansion before the transduction of the c-Myc, Kruppel-like factor 4 (KLF4), sex-determining region of Y chromosome-box 2 (SOX2) and octamer-binding transcription factor 4 (OCT4)-containing Sendai viruses for 3 days. After transfer into Geltrex, we monitored hiPSC clones for 30 days (Figure 1C). We confirmed pluripotency by demonstrating phosphatase alkaline activity, cell surface SSEA3/4 and TRA1-60 expression, and *OCT4*, *NANOG*, *SOX2* mRNA expression [20,21]. We assessed HiPSC genetic integrity by digital droplet PCR (iCS digital) (Supplementary Figure S2) [24]. One of the COPD-reprogrammed iPSC clones (iCOPD2) harbored a copy number gain in chromosome 20q11.21 at a late passage (>70 passages), yet differentiation could be achieved (Figure 1D). 

### 3.2. Cell Density and Induction Timing Are Critical for Successful Differentiation and Allows High Rate of Definitive Endoderm Induction

To develop a robust differentiation protocol (Figure 2A), we benchmarked the timing, cell density, and passaging method—three crucial steps for achieving reliable rates of DE purity and quality. We passaged hiPSC lines as single cells because hiPSC clumps were partly resistant to DE induction, as evidenced by OCT4 expression persistence. We obtained optimal cell adaptation by gentle colony dissociation into small clumps for five passages, and then into single cells for at least 5–10 passages, using Versene (EDTA) and Y-27632. Then, we started differentiation by adding activin A and CHIR99021 (a GSK3 inhibitor that acts as a WNT pathway agonist) in the presence of Y-27632 for 1 day (day 1; anterior primitive streak, (APS); Figure 2A,B and Supplementary Table S1), followed by activin A, LDN-193189 (a selective bone morphogenetic protein (BMP) signaling inhibitor that blocks the transcriptional activity of the type I BMP receptors activin receptor-like kinase 1, 2, 3, and 6), and Y-27632 for 1–2 days, leading to DE induction (day 2–3, Figure 2A,B). To optimize the protocol, we tested various intervals between hiPSC plating and APS induction, and different cell densities (from 70 to 130 K cells/cm^2^) (Figure 2C,D). Plating cells at too low and too high density led to important cell loss and to persistent OCT4 expression (Figure 2G). This optimized protocol robustly yielded >80% of C-X-C Motif Chemokine Receptor 4 (CXCR4) + DE cells within 2–3 days (Figure 2E) from the four hiPSC lines and one human ESC line (n = 170 independent experiments) (Figure 2F and Supplementary Figure S3). We did not observe any difference among cell lines at the DE stage. Moreover, DE cells expressed (mRNA level and protein) the characteristic endoderm transcription factors forkhead box A2 (FOXA2), SOX17, and the surface marker CXCR4 (Figure 2G–I). No difference was observed at the mRNA level between the healthy control and COPD cell lines.

### 3.3. Efficient Induction of High Purity NKX2.1+ Lung Progenitors without Cell Sorting

**Figure 3:**
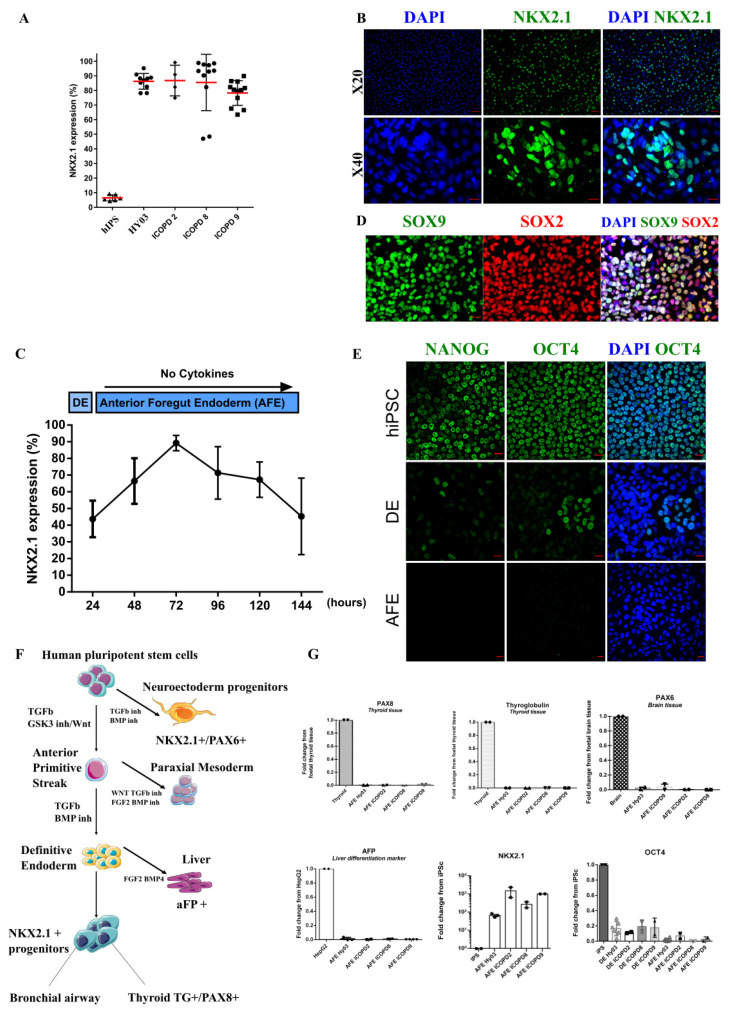
Anterior foregut endoderm characterization. (**A**) Percentage of NKX2.1+ cells after vAFE induction in the indicated cell lines. Undifferentiated hiPSCs: negative control. (**B**) Expression of NKX2.1, a ventral anterior foregut endoderm marker, assessed by immunofluorescence (HY03 cell line). Scale bar: 20 µm. (**C**) NKX2.1 expression kinetics (n = 3, HY03 cell line). (**D**) Expression of SOX2 and SOX9. Note the presence of SOX2/SOX9 double-positive cells (Hy03 cell line). (**E**) Analysis of the pluripotency markers NANOG and OCT4 in hiPSCs (top), and at the definitive endoderm (DE; middle) and ventral anterior foregut endoderm (AFE; bottom) stages (HY03 cell line); scale bar: 20 µm. (**F**) Model of hiPSC differentiation into the three embryonic layers, emphasizing that NKX2.1 is expressed by bronchial, neuroectodermal, and thyroid progenitors. These other progenitors can be a potential source of cell contamination in NKX2.1+ cells during iPSC differentiation into lung progenitors. Inh, inhibition. (**G**) Quantitative PCR analysis to assess contamination at the AFE stage by thyroid gland (thyroglobulin (*TG*) and paired box 8 (*PAX8*)), liver (alpha fetoprotein, *AFP*), and brain (paired box 6, *PAX6*) progenitors; gene expression was normalized to the tissue control (listed below). Progressive downregulation of the pluripotency marker *NANOG*. *NKX2.1* expression at the different differentiation stages was normalized to expression in iPSCs.

Comparison of various growth factor combinations for vAFE induction showed that DE cells needed minimal cell signaling, and therefore, were grown in RPMI1640 basal medium with B27 minus vitamin A (Figure 2A and Supplementary Tables S1–S3). For efficient vAFE induction, a DE cell population with at least 80% of CXCR4+ cells was required. Time course experiments showed that at 24–36 h after LDN-193189 addition, there was a narrow window when cells exhibited optimal conditions (i.e., high CXCR4 expression and high viability) for vAFE induction. The 3D bud-like structures emerging between days 4–8 appeared to be a good morphological indicator of vAFE differentiation visible under an optical microscope (Figure 2B, red arrows). In these conditions, >80% of cells consistently expressed NKX2.1 (assessed by flow cytometry and immunolabelling in five different PSC lines; n = 46 independent experiments) (Figure 3A,B, Supplementary Figure S3). The optimum percentage of NKX2.1+ cells (>80%) was reached at ~day 3 after vAFE induction (Figure 3C), as confirmed by immunostaining for NKX2.1 from day 1 to day 4 (Supplementary Figure S4A). This NKX2.1 expression level was required to induce differentiation towards iALI. Pluripotency markers (e.g., OCT4 and NANOG) were strongly downregulated at the vAFE stage, compared with the DE stage (Figure 3E–G). Positive Controls: Brain mRNA, Thyroid mRNA, HepG2 (Human Liver Cancer Cell Line) mRNA. 

NKX2.1 bronchial progenitor cells exhibited a high proliferation rate, assessed by quantifying the expression of the proliferation marker protein Ki-67 (Supplementary Figure S4C). We also detected SOX2, SOX9 expression by immunostaining, as previously reported in vivo during human lung development (Figure 3D, Supplementary Figure S4B), but not terminal airway epithelial markers. This confirmed the immature feature of these progenitor cells, and was in agreement with another hiPSC differentiation protocol [18] and human lung development [25]. As NKX2.1 is also expressed in other developing tissues (Figure 3F), we assessed by RT-qPCR, the purity of NKX2.1+ lung progenitor cells by confirming the absence of thyroid gland- (thyroglobulin, paired box 8), brain- (paired box 6), and liver-specific (alpha-fetoprotein, confirmed also by immunostaining in Supplementary Figure S4D) cell markers. We did not observe any difference between healthy and COPD cell lines at the bronchial progenitor stage.

### 3.4. Specification of NKX2.1 Lung Progenitor Cells in 2D ALI Culture Conditions Leads to Functional, Multi-Ciliated Airway Epithelium

We obtained iALI bronchial epithelium from four different hiPSC lines (n > 3 independent experiments per cell line). After mechanical dissociation into small clumps, we plated vAFE cells at high density on Transwell inserts in PneumaCult-Ex Plus medium (day 9, Figure 2A). After 2 days in PneumaCult-Ex Plus medium, we progressively switched to PneumaCult-ALI maintenance medium. Four days after seeding on Transwell inserts, we removed the medium from the apical side to switch to ALI culture (“polarization”). We added DAPT, a γ-secretase inhibitor that blocks NOTCH signal transduction, to the culture medium in the basolateral part of the Transwell from day 14 to day 28, post-plating on Transwell inserts (Figure 2A and Supplementary Table S1).

#### 3.4.1. Epithelium with Barrier Function

**Figure 4:**
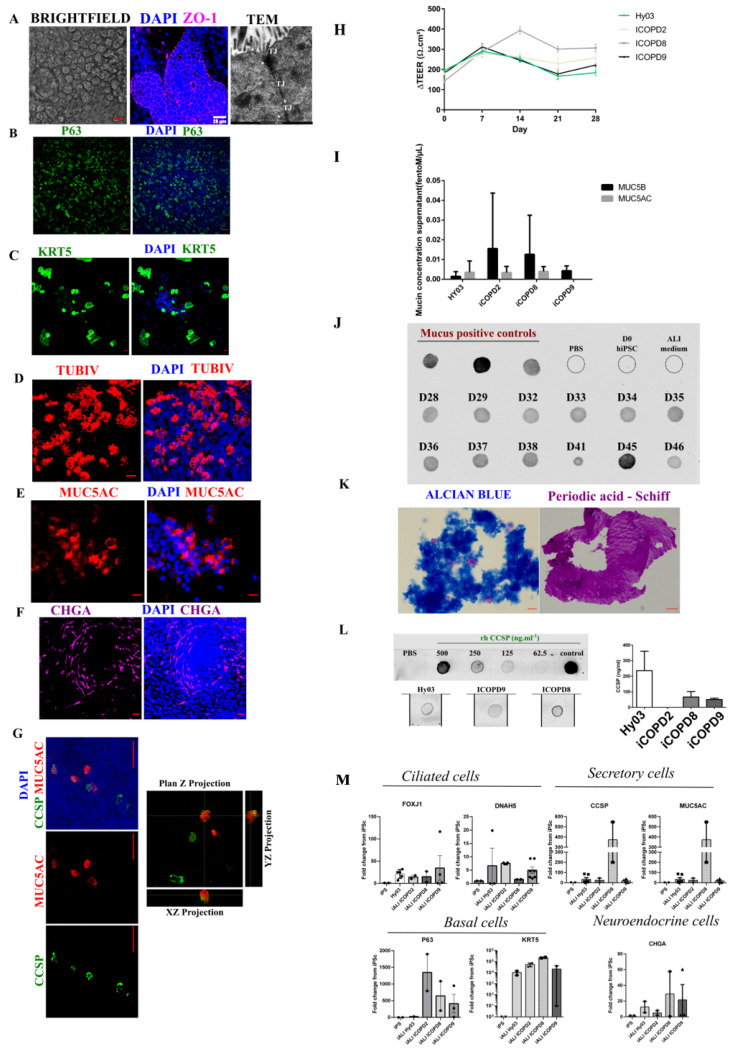
Human induced pluripotent stem cell (hiPSC)-derived bronchial airway epithelium at day 45 of differentiation (iALI). (**A**) Epithelial cells: optical microscopy image (left panel), immunolabeling of zonula occludens protein 1 (ZO-1) (middle panel); (right panel) transmission electron microscopy image of mature hiPSC-derived airway epithelium grown at the air–liquid interface (iALI) after 45 days of differentiation. The image shows two contiguous ciliated cells with ciliated structures at the apical side of the polarized iALI epithelium; epithelial features are highlighted by the presence of tight junctions (TJ) and desmosomes (white arrowhead) (iCOPD9 cell line). (**B**,**C**) Basal cells: TP63 (HY03 cells) and KRT5 (iCOPD9 cells) expression. (**D**) Multi-ciliated cells: expression of the terminal differentiation marker TUBIV (iCOPD2 cell line). (**E**) MUC5AC+ goblet cells (iCOPD9 cell line). (**F**) Rare clusters of CGHA+ neuroendocrine cells (iCODP9 cell line). (**G**) Left panels: immunostaining of CSSP+ club cells and MUC5AC+ goblet cells in cultures grown without DAPT. Note the presence of CCSP/MUC5AC double-positive cells (iCODP9 cell line). Right panels: Two-color confocal image showing CCSP (green) and MUC5AC (red) co-localization at day 14 of ALI culture. Orthogonal views (XY, XZ, YZ) showing colocalization (yellow) of CCSP and MUC5AC (iCDOP9 cell line). Scale bar: 20 µm. (**H**) Transepithelial Electrical Resistance (TEER) quantification at different time points in the four hiPSC lines at the iALI differentiation stage; day 0 is the day of ALI polarization. The electrical resistance of the blank inserts with medium alone was subtracted from the TEER values of co-cultures. Data represent the mean ± SD of at least three different experiments for each cell line, each with at least three TEER measurements. (**I**) MUC5AC and MUC5B concentration in supernatants from iALI cultures after 30 days of differentiation, assessed with the LC-MRM method. (**J**) Dot blot analysis to detect MUC5AC presence in supernatants of one iALI bronchial epithelium culture (derived from the HY03 cell line) from day 28 to day 46 of differentiation. Supernatant from an ALI culture of human bronchial epithelial cells from a healthy control was used as positive control of mucins. (**K**) Alcian blue and Periodic Acid–Schiff (PAS) staining of mucus in iCOPD9 culture supernatant. (**L**) CCSP quantification at day 45 in supernatants from iALI bronchial epithelium cultures derived from the HY03, iCOPD9, and iCOPD8 hiPSC lines. (**M**) Quantitative PCR analysis to assess the expression of *FOXJ1* and *DNAH5* (ciliated cells), *MUC5AC* and *CCSP* (secretory cells, and goblet cells and club cells, respectively), *P63* and *KRT5* (basal cells), and *CHGA* (neuroendocrine cells). Scale bar: 20 μm.

HiPSC-derived epithelial cells reached confluence after 4 days of submerged growth conditions (Figure 2A). We observed morphological features consistent with epithelium at late iALI stage (>day 42), zonula occludens 1 expression, and the presence of adherent junctions (junctional complexes) by transmission electron microscopy at day 34 (Figure 4A). We assessed the barrier integrity during ALI 2D culture by TEER measurement. TEER increased significantly during the differentiation process (Figure 4H). At day 7 of air liquid interface polarization, it reached ~300 Ω·cm^2^ and could be maintained for >200 days of culture. TEER values were not significantly different between control and COPD hiPSC-derived epithelia at all time points.

#### 3.4.2. IALI Bronchial Epithelium Includes Major and Rare Solitary Human Airway Epithelial Cells

At day 45 of differentiation, we could identify the main bronchial epithelium cell types: basal cells (keratin 5, KRT5, and tumor protein P63, TP63) (Figure 4B,C), ciliated cells (tubulin beta 4, TUBIV) (Figure 4D), goblet cells (mucin-5AC, MUC5AC) (Figure 4E), club cells (Secretoglobin family 1A member 1, CCSP, also known as SCGB1A1) (Figure 4G), and neuroendocrine cells (chromogranin A, CHGA) (Figure 4F). We detected club cells and goblet cells in iALI cultures already at day 27 of differentiation (Figure 4G,I). We detected CCSP+/MUC5AC- and CCSP-/MUC5AC+ cells, but also a small number of CCSP+/MUC5AC+ cells at day 27 of differentiation (day 14 after ALI polarization) (Figure 4G), as confirmed by confocal analysis. The presence of MUC5AC+ cells (Figure 4E–G) was associated with the protein release in the supernatant, detected by dot blot analysis, alcian blue and periodic acid–Schiff staining (Figure 4J,K). Quantification of the two main mucins, MUC5B and MUC5AC, in supernatants after day 45 of differentiation (at least n = 3 independent experiments for each cell line) showed that MUC5B was the predominant mucin secreted by healthy control (HY03 cells: 0.001 (0.7-4-0.004 fmol/µL) and COPD hiPSC-derived cultures (0.012 (0.8-4-0.06) fmol/µL) (Figure 4I). The mean MUC5AC concentration also was similar between COPD and healthy control hiPSC-derived cultures (0.003 (0–0.008) fmol/µL and 0.003 (0.5-4-0.01) fmol/µL).

The concentration of secreted CCSP ranged from 103.9 ng/mL to 110.9 ng/mL, depending on the experiment (Figure 4L), and was comparable among cell lines at day 41 of differentiation. We could not detect CCSP in the iCOPD2 cell line.

We also detected neuroendocrine cells (CHGA mRNA and protein expression) that could organize into clusters, resembling airway neuroepithelial bodies (Figure 4F–M). We did not detect SFTPC expression at mRNA level at iALI stage (Supplementary Figure S4E). 

#### 3.4.3. Functional Multi-Ciliated Cell Airway Epithelium

**Figure 5:**
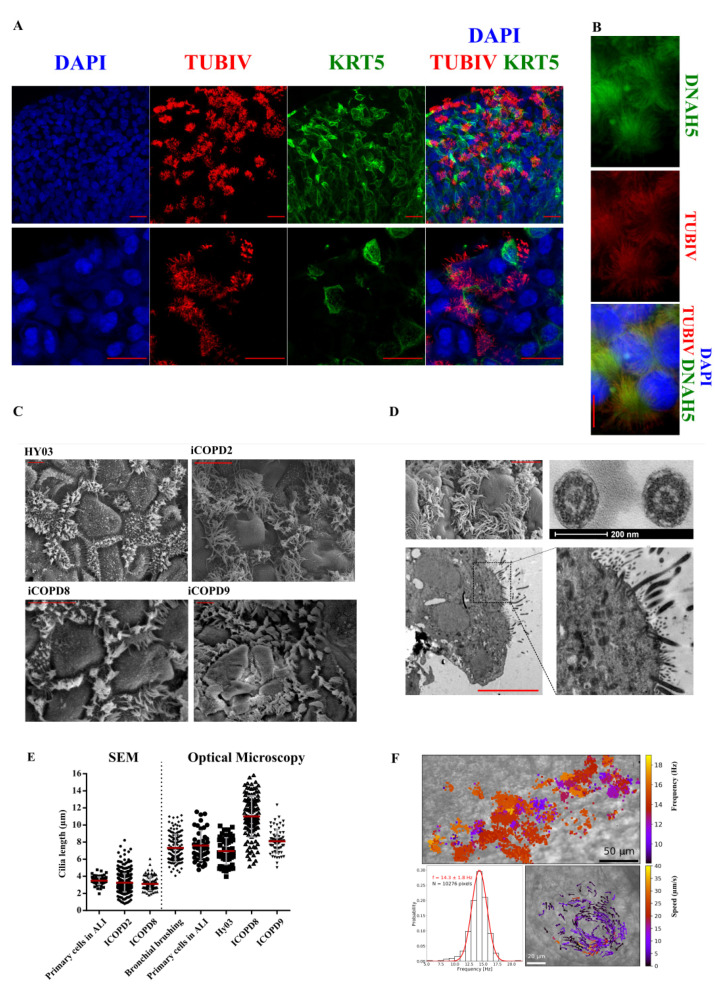
Multi-ciliated bronchial epithelium and cilia characterization at day 45 of differentiation. (**A**) Confocal microscopy images of TUBIV (ciliated cell marker) and KRT5 (basal cell marker) expression; iCOPD2 cell line. (**B**) Multi-ciliated cell characterization using anti-DNAH5 and -TUBIV antibodies. DNAH5 is localized in the axoneme in the iALI bronchial epithelium derived from the iCOPD9 cell line (upper panel). TUBIV is detected only in cilia (middle). Merging of the DNAH5 and TUBIV signals (lower panel); iCODP9 cell line. Scale bar: 10 µm. (**C**) Scanning electron microscopy images of hiPSC-derived iALI airway epithelium after 45 days of differentiation showing the presence of multi-ciliated cells in iALI epithelia derived from the indicated hiPSC lines. Scale bar: 10 µm. (**D**) Top left: Scanning electron microscopy (SEM) image of ciliated cells used for cilium length determination; iCOPD9 cell line. Scale bar: 10 µm. Right panels: cilium cross-sections by transmission electron microscopy. (**E**) Determination of cilium length by SEM (left) and optical microscopy (OM; right) in the different cell lines. Cilium length measurement was performed in primary cells in ALI (n = 91 by SEM and n = 45 by OM), bronchial brushing samples from patients with COPD (n = 141 by OM), iCOPD2 (n = 428 by SEM), iCOPD8 (n = 98 by SEM and n = 120 by OM), HY03 (n = 51 by OM), and iCOPD9 cells (n = 66 by OM). (**F**) Top: Ciliary beating frequency map from a movie (500 frames per second); iCOPD2 cell line. Scale bar: 50 µm. Bottom left: mean ciliary beating frequency distribution. Bottom right: vectors representing the orientation and celerity of the vortex flow generated by ciliary beating; iCOPD8 cell line. Scale bar: 20 µm. Hz = hertz.

We also observed cilia beating by optical microscopy and by TUBIV immunofluorescent labeling (Figure 5A). We identified abundant multi-ciliated cells in all four iPSC lines after 30 days of differentiation. We observed dynein axonemal heavy chain 5 staining along the ciliary axoneme (Figure 5B). Morphological analysis of multi-ciliated cells by optical and transmission electron microscopy (Figure 5C,D) indicated that the cilium structure was characterized by nine peripheral doublet pairs and a central pair of singlet microtubules (Figure 5D), typical of motile cilia [26].

We then measured cilium length in iALI cultures, in fresh bronchial epithelial cells obtained by endoscopic brushing, and in classical ALI-cultured airway epithelium by optical and scanning electron microscopy. The mean cilium length was similar in ALI and iALI cultures (Figure 5E), without any obvious difference among the different samples. We could observe cilia beating using a high-speed camera after isolation of iALI epithelium patches (Supplementary Movie S1), on Transwell membranes (Supplementary Movie S2), and after live immunostaining using SiR-conjugated fluorogenic probes for tubulin (Supplementary Movie S3). 

To assess the muco-ciliary clearance capacity of 2D cultures, we recorded CBF and muco-ciliary flow. The CBF of iALI cultures (14.3 ± 1.8 Hz) was similar to that of primary airway epithelium in ALI culture (Figure 5F) [27]. Cultures presented structures with high density of ciliated cells that were actively beating, giving rise occasionally to localized vortexes (Figure 5F, right bottom panel, Supplementary Movie S2). The estimated flow velocity of the vortex was 5.6 ± 6.5 μm/s. We could observe beating cilia in iALI bronchial epithelia for ~400 days without cell passaging and without aneuploidy appearance (Supplementary Figure S2B). Moreover, we could passage cultures at least three times after iALI generation. 

## 4. Discussion

Here, we described the generation of iALI bronchial epithelium that represents an attractive alternative to animal models and ex vivo cultures of differentiated bronchial epithelium from endobronchial biopsies. Our differentiation protocol offers a virtually unlimited source of homogeneous reliable human bronchial epithelium. Importantly, this protocol was carried out successfully by ten different members of our research group, and at least three times for each cell lines. 

In vitro models of human epithelia in ALI culture represent useful platforms to promote the differentiation and maturation of epithelial cells and allow the modeling of infections and environmental exposures. The generation of mature bronchial epithelium from hiPSCs is a powerful way to explore and recapitulate in vitro human airway development through a series of steps that mimic the normal in vivo embryonic development. Furthermore, iALI is an unlimited source of airway epithelium. HiPSC differentiation provides also a mean to characterize the different signaling pathways involved in airway lineage specification and differentiation [28]. Besides being an excellent tool for modeling human airway development, iALI represents an optimal platform for therapeutic innovation, extensive drug screening, and for cell-based therapy.

The limitations of our iALI system are mainly linked to the potential lack of purity of iPSC-derived airway progenitors and the difficulties to achieve fully matured airway epithelium. However, recent single cell transcriptomic analyses indicated that human airway primary cells from bronchial biopsies and adult human alveolar epithelium share a common signature with iPSC-derived lung epithelium [29,30]. Although iALI bronchial epithelium generation is slower than that of ALI epithelium obtained from airway tissue samples, it provides a potentially unlimited quantity of epithelium from a given donor, thus, avoiding batch heterogeneity due to multiple donors. 

We identified several critical factors that ensure the efficiency and reproducibility of airway epithelium differentiation from human PSCs. First, PSCs must be adapted to single-cell culture for homogenous cell seeding. When we tried to plate non-adapted cells (i.e., large clumps or high cell density), cell loss was reduced, but differentiation was hampered (Figure 2D,G). This could be explained by the sustained expression of pluripotency transcription factors within clumps and/or by altered YAP/TAZ signaling activity. Second, the homogeneity of DE and vAFE cell populations (CXCR4 and NKX2.1 expression in ≥80% of cells at the relevant step) was a good predictor of the final success. Based on the work by Matsuno et al. [17], we found that APS induction by activation of the activin A/nodal and WNT pathways for 24 h, followed by two additional days of activin A activity and TGFβ pathway inhibition for DE induction, without addition of other cytokines or small molecules during the vAFE stage, was an effective strategy. Both SOX2 and SOX9 were expressed at the vAFE stage with many double positive cells, in accordance with previous studies reporting the presence of these bipotent cells specifically in human PSC, further strengthening the iALI model [25]. To efficiently isolate NKX2.1+ bronchial progenitors during hiPSC differentiation, several cell surface molecules specifically expressed in these cells have been tested. Carboxypeptidase M (CPM), a specific marker of NKX2.1+ airway progenitors that generate type II alveolar epithelial progenitor cells, was proposed as a cell surface marker for sorting NKX2-1+ cells derived from human iPSCs [13,31]. However, CPM is strongly expressed in hepatoblasts and fetal liver progenitor cells and is present during the hepatic specification of iPSC-derived endoderm cells. This may limit its use for sorting lung progenitor during endoderm cell differentiation [18,32]. Hawkins et al. reported that sorting CD47^high^CD26^low^ cells allowed enriching the NKX2-1+ lung progenitor population from 62% to 70%. Therefore, improving NKX2-1+ lung progenitor sorting based on cell surface markers could help to refine our differentiation strategy. Nevertheless, we found that this step was not necessary for robust bronchial epithelium induction, thus, overcoming a major bottleneck of directed differentiation protocols.

Another key point was the use of the PneumaCult differentiation medium. This proprietary medium, the composition of which is not disclosed, efficiently promotes the differentiation of primary cells obtained from bronchial biopsies. Although this medium might contain a NOTCH pathway inhibitor, we added DAPT to our differentiation protocol. Indeed, NOTCH signaling inhibition promotes the differentiation into multi-ciliated cell at the expenses of club cells [33]. This protocol generated epithelia containing CCSP+/MUC5AC+, CCSP+/MUC5AC-, and CCSP-/MUC5AC+ cells, although basal and ciliated cells were predominant. Interestingly, we detected also rare cells, such as chromogranin A-expressing neuro-endocrine cells. Altogether, these features suggest that the generated epithelia reproduced many features of a fully differentiated bronchiolar epithelium [34]. The physiological relevance of the model was reinforced by the detection of plugs of mucus (alcian blue and periodic acid–Schiff staining), the formation of vortexes of muco-ciliary clearance, cilium length, and CBF, as observed in vivo.

Besides its reproducibility and simplicity, our protocol provides a 2D bronchial epithelium, unlike other methods that lead to 3D ciliated organoids [12,14,15]. To the best of our knowledge, these three COPD hiPSC lines are the first described in the literature, although difficulties could have been expected given the previously reported relative CD34 deficiency [35]. Moreover, one COPD hiPSC-derived epithelium culture could be kept consistently differentiated for ~400 days at the time of writing. As expected for a disease with multifactorial genetic susceptibility to environmental triggers (e.g., cigarette smoke), and considering that cell reprogramming erases most epigenetic marks, the DE cells and iALI bronchial epithelia derived from the COPD hiPSC lines were mostly identical to those derived from the healthy donor. One notable difference was basal MUC5B secretion that was increased in all iALI bronchial epithelia derived from COPD hiPSC lines. It will be interesting to challenge these iALI epithelia with smoke extract or pollution particles and investigate whether mucins are induced, as observed in smokers and patients with COPD [36,37].

## 5. Conclusions

In conclusion, we described an easy and reliable method to drive PSC differentiation into 2D multicellular bronchial epithelium. This method is highly reproducible, efficient, does not require cell sorting, and is achievable using blood cells from patients with polygenic lung diseases. Our protocol recapitulates the generation of bronchial airway during lung development, particularly the distal bronchial pattern. The protocol will also allow the studying of chronic airway diseases, especially those that concern mainly the small airways, such as cystic fibrosis, COPD, severe asthma, and idiopathic pulmonary fibrosis [38].

## Data Availability

Not applicable.

## Supplementary Materials

The following supporting information can be downloaded at: https://www.mdpi.com/article/10.3390/cells11152422/s1, Figure S1: Clinical characteristics of patients with COPD; Figure S2: Genetic integrity of the hiPSC lines used for differentiation into iALI bronchial epithelia; Figure S3: NKX2.1 and CXCR4 FACS gating strategy; Figure S4: Characterization of vAFE progenitors: SOX9 expression and NKX2.1 expression changes during v-AFE induction; Table S1: Medium composition by culture period; Table S2: Molecules and used concentration; Table S3: List of reagents and consumables; Table S4: List and sequences of the primers used for RT-qPCR; Movie S1: iALI bronchial epithelium obtained from the iCOP9 hiPSC cell line (X20), Movie S2: iALI bronchial epithelium obtained from the iCOPD8 hiPSC cell line (X40); Movie S3: iALI bronchial epithelium obtained from the iCOPD9 hiPSC cell line after live immunofluorescence for TUBIV [39,40]

## Appendix A

Supplementary clinical data: supplementary information of COPD history.

### COPD Patients’ Characteristics and History

The healthy control (41 years at inclusion) had normal lung function, without respiratory symptoms from childhood, and had no family history of chronic airway diseases.

Patients with severe COPD were recruited in the framework of the INVECCO project. Patients had normal blood level of α-1 anti-trypsin and none of them carried any TERT mutations, to avoid any monogenic COPD form.

Early disease onset was suggested by the early appearance of symptoms, at the mean age of 35 years.

Disease was revealed by dyspnea, pneumothorax, or acute exacerbation. The mean time between first symptoms and COPD diagnosis was 12 years. Family history of COPD (first-degree relative) was found for two of the three enrolled patients. All of them were at least GOLD stage 3. Environmental exposures were identified for all three patients: early and heavy active tobacco exposure, in utero exposure to tobacco, and to second-hand smoke in early childhood. Their mean cigarette consumption was 50 pack-years (range, 30–75). Consumption of cannabis and intravenous heroin was recorded, in agreement with the already reported association between substance consumption and severe emphysema. Dyspnea was severe, requiring long-term home oxygen support for two patients. No cardiovascular comorbidity, diabetes, cancer, or pulmonary hypertension was described, but they had severe osteoporosis despite their young age. Two of the three patients with COPD had recurrent spontaneous pneumothorax. Two patients had three or more exacerbations per year that required hospitalization. All three patients received at baseline long-acting beta2-agonist (LABA) and anti-cholinergic (LAMA) drugs, but not inhaled corticosteroid (ICS). Chest CT imaging showed severe apical centrilobular emphysema, basal bronchiectasis, and increased wall thickness (Figure 1A, left panel). Lung function declined fast in these patients with COPD. The mean change in FEV1 (mL/year) was −25.3 (SD, 43.3) mL/year (Figure 1A, right panel).

At the time of inclusion, all three patients with COPD were on the lung transplantation waiting list. At the time of manuscript submission, patient iCOPD2 was admitted in the intensive care unit for a severe COPD exacerbation that required mechanical ventilation. Patient iCOPD8 refused lung transplantation. Patient iCOPD9 was programmed for single lung transplantation (pre-transplant pleurodesis due to iterative pneumothorax). The lung transplant was delayed for more than 24 months due to overweight. Recently, a localized lung adenocarcinoma was discovered. Bronchoscopic lung volume reduction has been proposed to this patient due the severe emphysema phenotype.
