# Supplementary material for: Differentiation of Human Induced Pluripotent Stem Cells from Patients with Severe COPD into Functional Airway Epithelium
Source: Cells. 2022 Aug 5;11(15):2422. doi: 10.3390/cells11152422 (PMC9368529; doi:10.3390/cells11152422)
Supplement: Supplementary file 1 [file cells-11-02422-s001.zip › Bourdin De Vos Supplemental Figure S1.pdf]

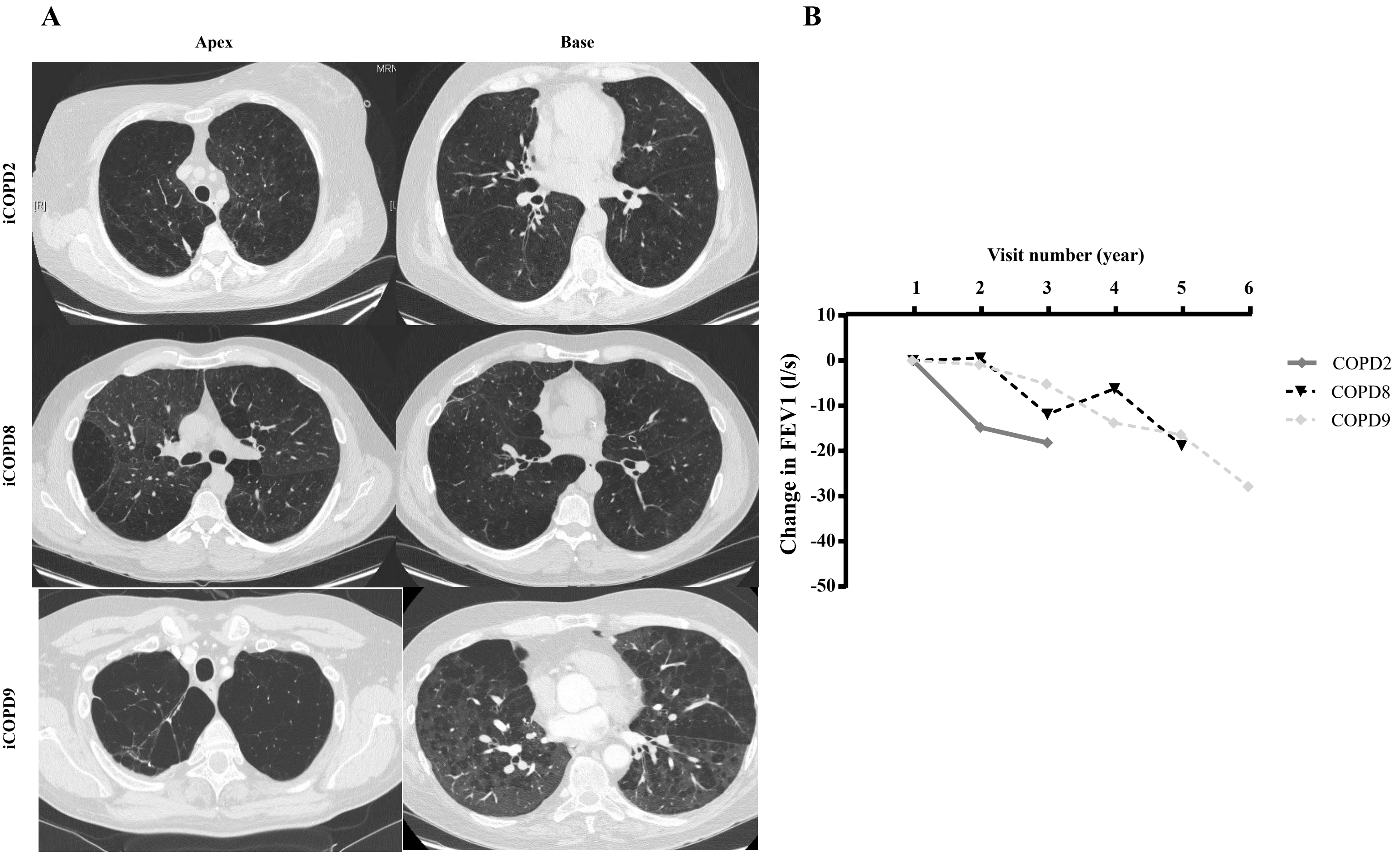

C : Baseline characteristics of COPD patients

	COPD2	COPD8	COPD9
Age (years)	52	46	54
Sex	F	M	M
Body-mass index (kg/m2)	30	24	32
**Smoking**			
Current/ Ex-smoker	Current	Ex-smoker	Ex-smoker
Pack-year history	45	30	75
Age of beginning (years)	14	18	24
**Others toxic ***	Yes	Yes	No
**Pulmonary function and symptoms**			
Age of diagnosis	48	43	49
Age at onset of first symptoms (years)	40	30	35
In utero smoking exposure	Yes	Yes	Yes
Premature birth	No	No	Yes
Familial history of CODP/emphysema	Yes	No	Yes
Increased dyspnea	Yes	Yes	Yes
Increased cough	Yes	No	Yes
Medical Research Council dyspnea scale	4	1	4
**Lung function**			
FEV (L)	0.35	1.19	0.97
FEV (%)	14	34	30
FEV1/FVC ratio (%)	32	25	36
RV (L)	4.39	3.21	4.72
RV (%)	244	156	213
DLCO (%)	30	41	35
**Medication**			
Long acting β2 agonist	Yes	Yes	Yes
Long-acting muscarinic antagonist	Yes	Yes	Yes
Inhaled corticosteroid	No	No	No
Use of benzodiazepine	Yes	Yes	Yes
Azithromycin	Yes	No	No
**Clinical findings**			
Oxygen supply (L/min)	1	0	3
BODE index	6	3	6
Exacerbation rate in previous 12 months	3	3	1
Pneumothorax	No	Yes	Yes
Osteoporosis	Yes	No	Yes
Pulmonary hypertension**	No	No	No
Coronary Artery Disease	No	No	No
Lung cancer	No	No	No
Type 2 diabetes	No	No	No
GORD	Yes	Yes	Yes
Anxiety/Depression	Yes/No	Yes/Yes	Yes/Yes
**Blood test**			
Eosinophils (10^9 cells per L)	150	N.A	810
Eosinophils (% total WBC)	2.4	N.A	5.9
α1 anti trypsin (g/l)	1.16	1.37	1.1
Pa02 (mmHg, ambiant air)	62	82	76.6
PaC02 (mmHg, ambiant air)	48.8	32.6	44.4
**CT scan**			
Emphysema (wall lung) %	18.4	12.99	31.95
WA ratio mean (min-max)	59.75 (48.1-68.1)	57 (44.9-69)	68.2 (57.7-77.1)
