# Supplementary material for: Differentiation of Human Induced Pluripotent Stem Cells from Patients with Severe COPD into Functional Airway Epithelium
Source: Cells. 2022 Aug 5;11(15):2422. doi: 10.3390/cells11152422 (PMC9368529; doi:10.3390/cells11152422)
Supplement: Supplementary file 1 [file cells-11-02422-s001.zip › Legends Supplementary Figures -- Cells.pdf]

**Supplemental Figure S1. Clinical characteristics of patients.**

(A) COPD patients. Left panel: high-resolution inspiratory CT images showing apical centrilobular, para-septal severe emphysema (column apex). In patients COPD2 and COPD8, bronchiectasis and increased airway wall thickness could also be observed (column base). (B) Right panel: rate of change in forced expiratory volume in 1 second (FEV1) over the years since diagnosis. Loss of lung function (% change from baseline) seems more accelerated in COPD patients in this study, around 20 to 30% during the follow up. The mean rate of FEV1 decline in iCOPD2, iCOPD8 and iCOPD9 was respectively 40 mL/year, 83 mL/year, 65 mL/year. (C) Baseline characteristics of COPD patients.

COPD = chronic obstructive pulmonary disease. FVC = forced vital capacity. FEV1 = forced expiratory volume, RV= residual volume. GORD = gastro-oesophageal reflux disease, Pa02 = partial pressure of oxygen. WA ratio= Wall Area ratio. Wall thickness was expressed as a ratio of the wall thickness to the total airway diameter (WA ratio) and mean value was calculated for each patient from all the bronchi measured. In this study, quantitatively assessment of emphysema was assessed by the percentage of low attenuation area (LAA%) divided by lung or lobe volume(s). A threshold of - 950 Hounsfield Units (HU) was used.

*: other substance abuse included cannabis, intravenous heroin, Subutex misuse (patient COPD2), and cannabis (patient COPD8).

**: Pulmonary hypertension was diagnosed on transthoracic echocardiography; if abnormal, right heart catheterization was performed.

**Supplemental Figure S2: Genetic integrity of the hiPSC lines used for differentiation into iALI bronchial epithelia**

(A) Genomic integrity evaluation of the hiPSC lines using the iCS-digital test [18]. Copy number variation analysis using droplet digital PCR and DNA extracted from the different hiPSC lines in culture (iCOPD8, iCOPD9, iCOPD2, PCD02.30 and HY03). All the hiPSC lines remained euploids, except iCOPD2 (clone A13) that displayed a copy number gain on chromosome 20q at mechanical passage 70 and clumps passage 3 (M70CL3) and was therefore later discarded. Error bars indicate the Poisson distribution (95% confidence intervals).

(B) Same analysis, using the iCS-digital Aneuploidy test to screen the 23 chromosomes in one iALI bronchial epithelium culture that was maintained for twelve months in culture.

**Supplemental Figure S3 NKX2.1 and CXCR4 FACS gating strategy**

**(A)** Gating strategy for the isolation of CXCR4 positive cells.

Flow cytometry gating strategy to viable CXCR4 cell subsets at the Definitive endoderm stage. Staining of single-cell solutions using isotype and CXCR4 conjugated antibodies and analysis by flow cytometry. (A-C) Gating strategy to exclude doublet (B) and isolate real single-cell unit (C). Among single cells, live cells were selected on absence of Zombie violet staining (D). (E) CXCR4 expression of cells compared to isotype control on PE staining **(B)** Gating strategy for the isolation of NKX2.1 positive cells.

(A) Flow cytometry gating strategy to viable NKX2.1 cell subset at the Anterior Foregut Endoderm stage. Staining of single-cell solutions using different unconjugated antibody and analysis by flow cytometry. (A-C) Gating strategy to exclude doublet and isolate real single-cell unit. (D) Among single cells, live cells were selected on absence of Zombie violet staining (E) NKX2.1 expression of cells compared to isotype control on Alexa 488 staining.

**Supplemental Figure S4. Characterization of v-AFE progenitors SOX9 expression and time course expression of NKX2.1 during v-AFE induction.**

(A) Kinetic expression of NKX2.1 by immunostaining during v-AFE induction, in HY03 cell line. Note increasing expression during the time course. Scale bar: 20µm.

(B) Immunostaining of HY03 -derived v-AFE cells for SOX9 (green) nuclear proteins. during v-AFE stage. Scale bars: 20 µm.

(C) Immunolabelling of HY03 cell line v-AFE stage for NKX2.1 (orange) and ki67 (green). Nuclei counterstained with DAPI.

(D) Immunofluorescence of v-aFE stage showing no contamination by AFP positive (liver) cells (PCD02.30 cell line). These results were available for all the others iPSC cell lines (data not shown). HepG2 hepatoma cell line was used as positive control, characterized by AFP expression (red). Scale bar 50µm.

(E) Quantitative PCR analysis to assess expression of SFTPC. Data represent the mean expression of STFPC in iCOPD9 cell line at Definitive Endoderm (DE) stage and iALI stage (n=2 independent experimental data points). Foetal lung was used as positive control.

**Supplemental Movie S1: iALI bronchial epithelium obtained from the iCOP9 hiPSC cell line (X20)**

Cilia beating was clearly visible on this video from iCOPD9 at Day 45. Acquisition was performed with inverted microscopy during cell culture routine check-up. The video represents cells that have been dissociated, before passaging cells into another coated Transwell. Dissociation was performed using Trypsin 10 minutes at 37°.

**Supplemental Movie S2: iALI bronchial epithelium obtained from the iCOPD8 hiPSC cell line (X40)**

This acquisition was used for biophysics analysis i.e. Ciliary Beat Frequency (CBF).

The video was recorded at 500 frames per second. This video includes 1500 frames.

**Supplemental Movie S3: iALI bronchial epithelium obtained from the iCOPD9 hiPSC cell line and with live immunofluorescence for TUBIV**
