# Supplementary material for: Differentiation of Human Induced Pluripotent Stem Cells from Patients with Severe COPD into Functional Airway Epithelium
Source: Cells. 2022 Aug 5;11(15):2422. doi: 10.3390/cells11152422 (PMC9368529; doi:10.3390/cells11152422)
Supplement: Supplementary file 1 [file cells-11-02422-s001.zip › Supplemental Table S1 vf.pdf]

Supplemental Table S1: Media composition by culture period

Media	Induced Pluripotent Stem cells	Anterior Primitive Streak	Definitive Endoderm	Anterior Foregut Endoderm	Lung Progenitor	Lung Airway Epithelium
**Days**	Day 0	Day 1	Day 2-3	Day 4-8	Day 9-10	Day 11-42+
**Basal media**	Essential 8 Medium<br>Penicillin-Streptomycin (10,000 U/mL) 1%	RPMI 1640<br>1x B27 supplement (50x), minus vitamin A<br>Penicillin-Streptomycin (10,000 U/mL) 1%	RPMI 1640<br>1x B27 supplement (50x), minus vitamin A<br>Penicillin-Streptomycin (10,000 U/mL) 1%	RPMI 1640<br>1x B27 supplement (50x), minus vitamin A<br>Penicillin-Streptomycin (10,000 U/mL) 1%	PneumaCult-Ex Plus Medium*<br>Penicillin-Streptomycin (10,000 U/mL) 1%	PneumaCult-ALI Medium**<br>Penicillin-Streptomycin (10,000 U/mL) 1%
**Add the day of use**	+ 10µM Y-27632	+ 10µM Y-27632<br>+ Activin A 100ng/ml<br>+ CHIR99021 3µM	+ 10µM Y-27632<br>+ Activin A 100ng/ml<br>+ LDN-193189 250nM	No cytokines	No cytokines	10 µM DAPT from day 28 to day 42
