# Supplementary material for: Differentiation of Human Induced Pluripotent Stem Cells from Patients with Severe COPD into Functional Airway Epithelium
Source: Cells. 2022 Aug 5;11(15):2422. doi: 10.3390/cells11152422 (PMC9368529; doi:10.3390/cells11152422)
Supplement: Supplementary file 1 [file cells-11-02422-s001.zip › Supplemental Table S2 vf.pdf]

Supplemental Table S2: Molecules and used concentration

Growth Factor	Stock Concentration	Final Concentration
**Activin A**	50 µg/ml	100 ng/ml
**Y-27632**	5 mM	10 µM
**CHIR99021**	3 mM	3 µM
**LDN-193189**	450 µM	250 nM
**DAPT**	10 mM	10 µM
