# Supplementary material for: Differentiation of Human Induced Pluripotent Stem Cells from Patients with Severe COPD into Functional Airway Epithelium
Source: Cells. 2022 Aug 5;11(15):2422. doi: 10.3390/cells11152422 (PMC9368529; doi:10.3390/cells11152422)
Supplement: Supplementary file 1 [file cells-11-02422-s001.zip › Supplemental Table S3 reagents vf.pdf]

Supplemental Table S3: list of reagents and consumables

REAGENT	SOURCE	IDENTIFIER
Chemicals, Peptides, and Recombinant Proteins		
Geltrex LDEV-Free, hESC-Qualified, Reduced Growth Factor Basement Membrane Matrix	Thermo Fisher	A1413301
StemSpan SFEM II	Stemcell	09605
StemSpan Erythroid Expansion Supplement	Stemcell	02692
Sodium Butyrate,	Stemcell	72242
CytoTuneTM −iPS 2.0 Sendai Reprogramming Kit	Thermo Fisher	A16517
B27 supplement (50x), minus vitamin A	Thermo Fisher	12587010
Y-27632 dihydrochloride	Tocris	1254
CHIR-99021	Sigma	SML1046-5MG
LDN-193189	Miltenyi biotec	130-103-925
DAPT	Tocris	2634/10
Activin A	Peprotech	AF-120-14E
Permeabilization Buffer (10X)	Thermo Fisher	00-8333-56
Triton X-100 (laboratory grade)	Sigma	X100-5ML
Bovine Serum Albumin	Sigma	A7906-100G
ProLong Gold Antifade Mountant	Thermo Fisher	P36930
Donkey serum	Sigma	D9663-10ML
Goat serum	Sigma	G9023-5ML
DAPI	Sigma	D9542-5MG
CryoStor CS10	Stemcell	07930
Zombie Violet Fixable Viability Kit	BioLegend	423113
Penicillin-Streptomycin (10,000 U/mL)	Thermo Fisher	15140122
Versene Solution	Thermo Fisher	15040033
Glutaraldehyde (25%)	Deltamicroscopies	16210
Paraformaldehyde (10%)	Deltamicroscopies	15712
Oligonucleotide primers	Integrated DNA Technologies (IDT)	See Table S4 - List and sequences of the primers used for RT-qPCR
RNA controls		
Human lung	Invitrogen	QS0618
Human thyroid	Invitrogen	QS0631
Human heart	Invitrogen	QS0614
Human colon	Invitrogen	QS0613
Human liver	Invitrogen	QS0617
Human brain	Invitrogen	QS0611
Antibodies (dilution)		
Donkey anti-rabbit IgG AlexaFluor 488 *(1:1000)*	Life technologies	A21206
Donkey anti-mouse IgG AlexaFluor 555 *(1:1000)*	Life technologies	A31570
Donkey anti-goat IgG AlexaFluor 647 *(1:1000)*	Life technologies	A21447
Mouse anti-CXCR4 (PE) *(1:200)*	BD Biosciences	557145
Mouse IgG2a, (PE) *(1:200)*	BD Biosciences	556653
Mouse anti-AFP *(1:200)*	Sigma	A8452
Mouse anti-Mucin 5AC *(1:200)*	Abcam	ab3649
Mouse anti-SOX2 *(1:100)*	Abcam	ab79351
Rabbit anti-SOX9 *(1:1000)*	Abcam	ab185230
Mouse anti-β-Tubulin IV *(1:200)*	Sigma	T7941
Rabbit anti-DNAH5 *(1:200)*	Sigma	HPA037470
Rabbit anti E-cadherin *(1:200)*	Santa Cruz Biotechnology	sc-7870
Rabbit anti-Oct 4 *(1:250)*	Santa Cruz Biotechnology	sc-9081
Rabbit anti-NANOG *(1:200)*	Abcam	ab80892
Rabbit anti-CCSP *(1:1000)*	BioVendor	Rd181022220
Rabbit anti-TTF1 (NKX2.1) *IF: 1:200 / FCM 1:1000*	Abcam	ab76013
Mouse anti-Chromogranin A *(1:200)*	Abcam	ab715
Rabbit anti-Ki67 *(1:200)*	Abcam	ab15580
Goat anti-SOX17 *(1:150)*	R&D Systems	af1924
Goat anti-FOXA2 *(1:150)*	R&D Systems	af2400
SiR-tubulin	Spirochrome AG	SC002
Software and Algorithms		
GraphPad	Prism	Version 6.01
ImageJ	National Institutes of Health	Version 1.52i
Inkscape	Free Software Foundation	Version 3
Alphaview sofware	Protein simple	Version 3.4.0.0
Zen 2.3	Carl Zeiss Microscopy	Version 4.03
Kaluza Analysis	Beckman Coulter	Version 2.1
Kaluza for Gallios Acquisition	Beckman Coulter	Version 1.0
Flow Cytometer (FCM)	Beckman Coulter	Gallios
Confocal Microscopy	Zeiss	LMS700

Odyssey imaging system	LI-COR	Odyssey 9120 Model
Scanning electron microscopy	Hitachi	S4000
Optical microscopy	Leica	DMi1 Model/MC170HD camera
Others		
Essential 8 Medium	Thermo Fisher	A1517001
DMEM/F-12, GlutaMAX Supplement	Thermo Fisher	31331093
RPMI 1640 Medium	Thermo Fisher	21875034
PneumaCult-Ex Plus Medium	Stemcell	05040
PneumaCult-ALI Medium	Stemcell	05001
PBS, pH 7.2	Gibco	20012019
SuperScript™ First-Strand Synthesis System	Invitrogen	11904-018
RNeasy Micro Kit	Qiagen	74004
RNeasy Mini Kit	Qiagen	74106
LightCycler® 480 SYBR Green I Master	Roche	04707516001
Alkaline Phosphatase Staining Assay (Red)	ScienCell Research Laboratories	8288
Alcian blue solution	Merck Millipore	1016470500
Periodic Acid – Schiff kit	Merck Millipore	1016460001
0.4 µm Pore Polyester Membrane Insert (Ø12 mm)	Corning	3460
TEER device	WPI	EVOM2 Model/ STX2 electrode
