# Supplementary material for: Differentiation of Human Induced Pluripotent Stem Cells from Patients with Severe COPD into Functional Airway Epithelium
Source: Cells. 2022 Aug 5;11(15):2422. doi: 10.3390/cells11152422 (PMC9368529; doi:10.3390/cells11152422)
Supplement: Supplementary file 1 [file cells-11-02422-s001.zip › Supplemental Table S4 primers vf.pdf]

Supplemental Table S4: list of PCR primers

Name	Size	Sequences
GAPDH_F	22	GAC CTG ACC TGC CGT CTA GAA A
GAPDH_R	21	CCT GCT TCA CCA CCT TCT TGA
OCT4_F	22	GGG CTC TCC CAT GCA TTC AAA C
OCT4_R	22	CAC CTT CCC TCC AAC CAG TTG C
NANOG_F	21	TGA TTT GTG GGC CTG AAG AAA
NANOG_R	21	GAG GCA TCT CAG CAG AAG ACA
AFP_F	25	CTA CCT GCC TTT CTG GAA GAA CTT T
AFP_R	22	GAT CGA TGC TGG AGT GGG CTT T
TG_F	20	ACG GTT CCT CGC AGT TCA AT
TG_R	20	GCA GCT TGG AAC ATA GGG GT
CDX2_F	22	ACA GTC GCT ACA TCA CCA TCC G
CDX2_R	22	CCT CTC CTT TGC TCT GCG GTT C
PAX6_F	19	TCT TTG CTT GGG AAA TCC G
PAX6_R	21	CTG CCC GTT CAA CAT CCT TAG
CHGA_F	20	CGG ATC CTT TCC ATT CTG AG
CHGA_R	20	ACC GCT GTG TTT CTT CTG CT
SFTPB_F	22	TCT GAG TGC CAC CTC TGC ATG T
SFTPB_R	22	TGG AGC ATT GCC TGT GGT ATG G
FOXJ1_F	22	GAG ACA GGT TGT GGC GGA TTG A
FOXJ1_R	22	ACT CGT ATG CCA CGC TCA TCT G
MUC5AC_F	20	CAT CTG CCA GCT GAT TCT GA
MUC5AC_R	20	AAG ACG CAG CCC TCA TAG AA
CCSP_F	20	CAT GAA ACT CGC TGT CAC CC
CCSP_R	20	GAT GAC ACG CTG AAA GCT CG
