# Supplementary figures and images for: Differentiation of Human Induced Pluripotent Stem Cells from Patients with Severe COPD into Functional Airway Epithelium
Source: Cells. 2022 Aug 5;11(15):2422. doi: 10.3390/cells11152422 (PMC9368529; doi:10.3390/cells11152422)

A

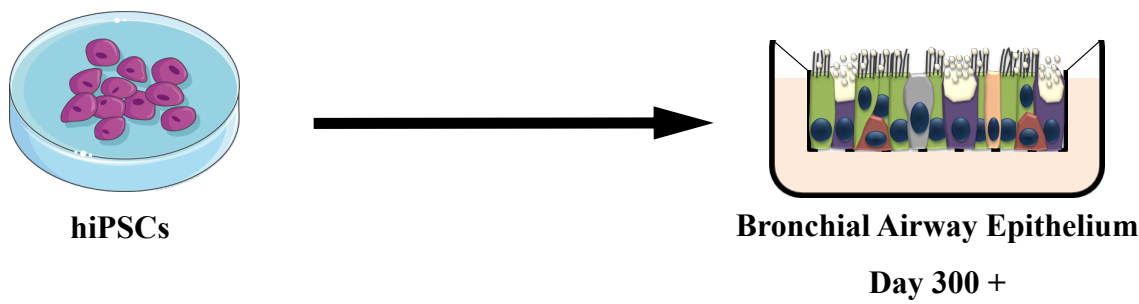

B

iCS- digital test

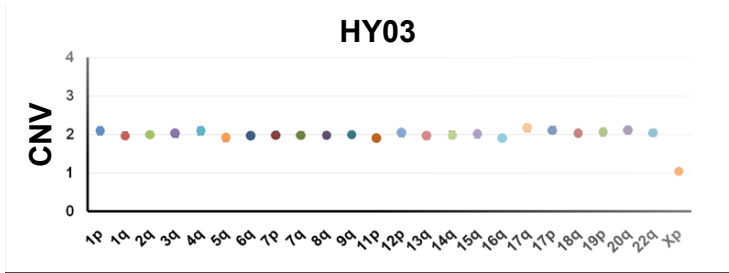

iCS-digital aneuploidy

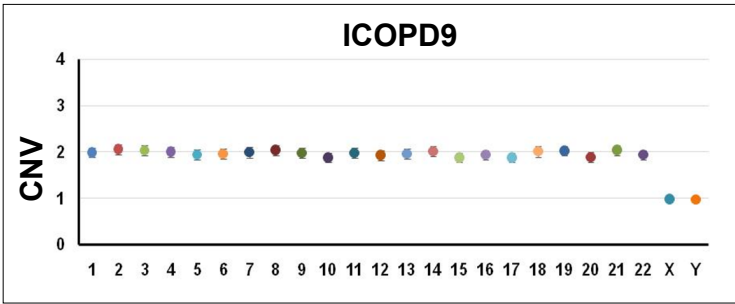

ICOPD8

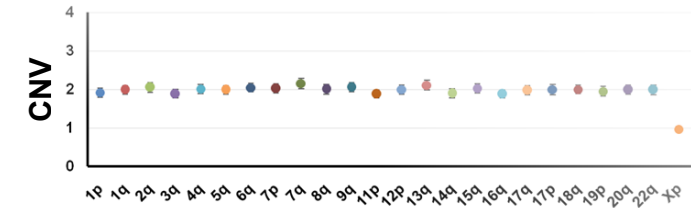

ICOPD9

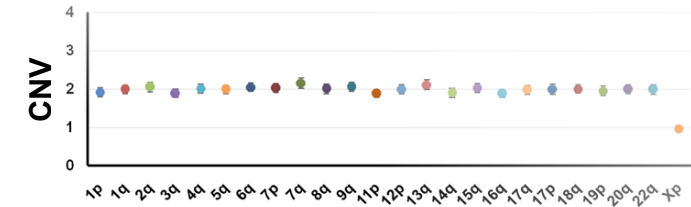

ICOPD2

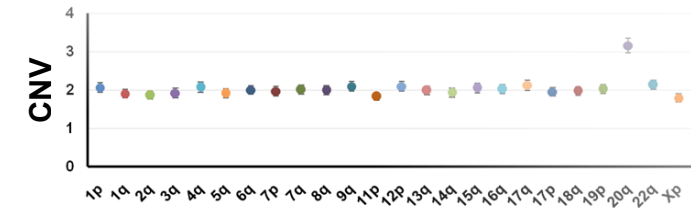

Supplement: Supplementary file 1 [file cells-11-02422-s001.zip › Bourdin De Vos Supplemental Figure S2.pdf]

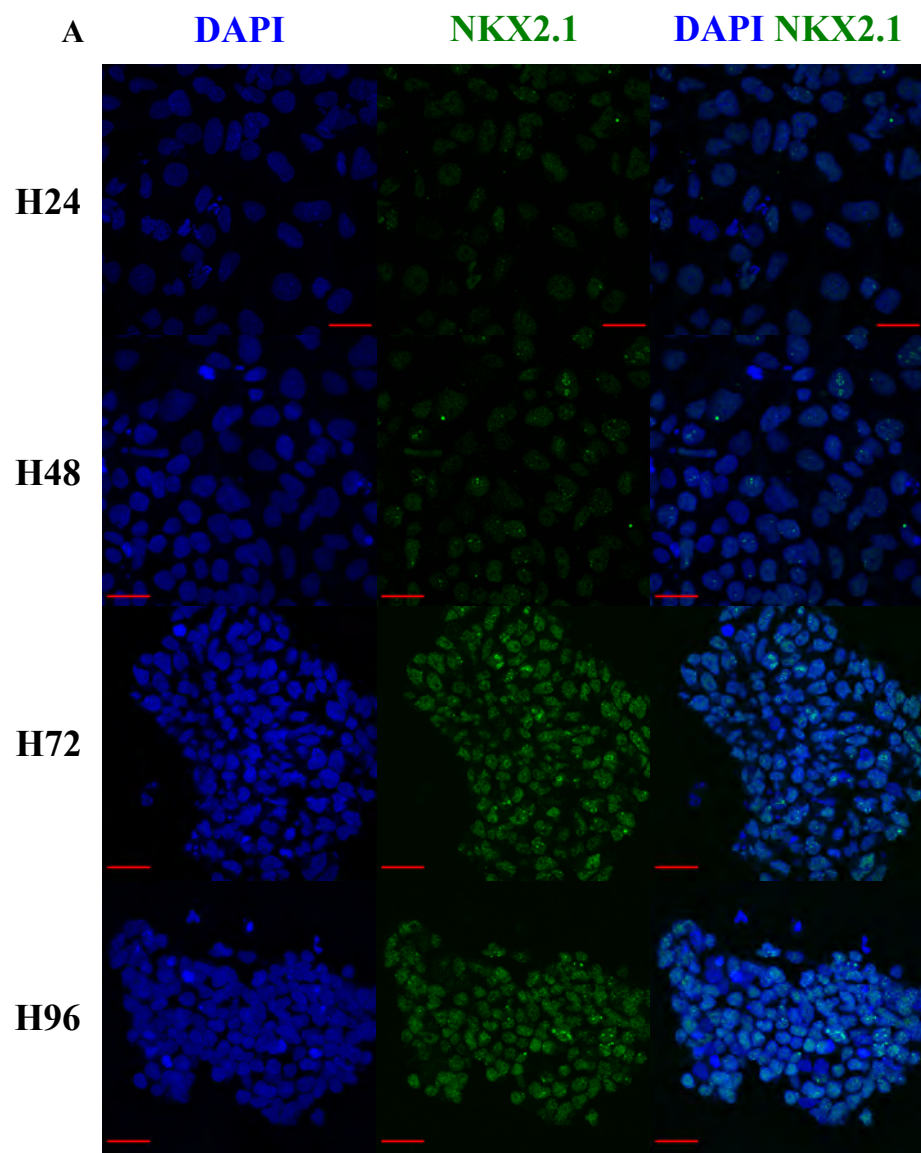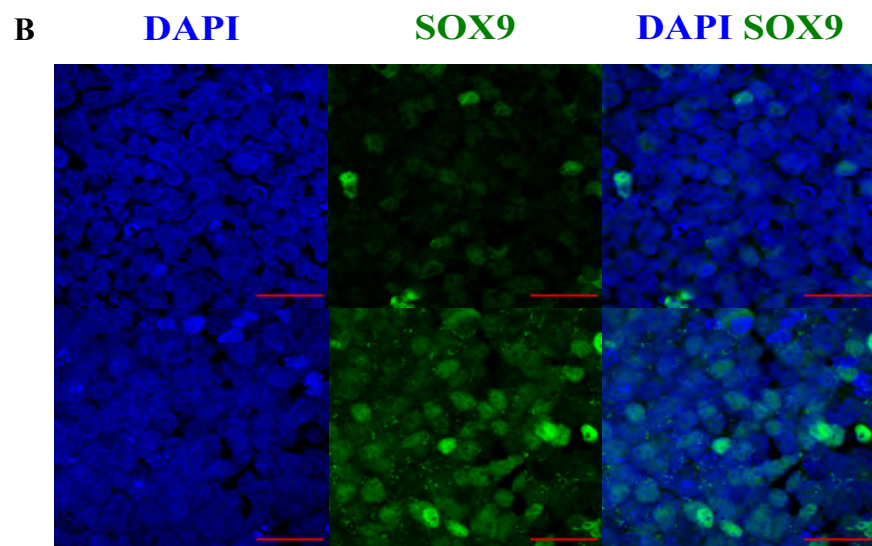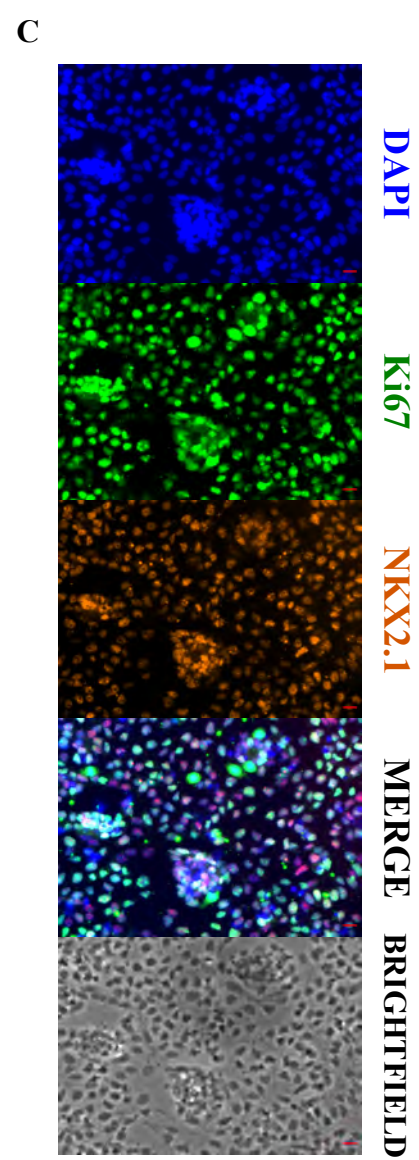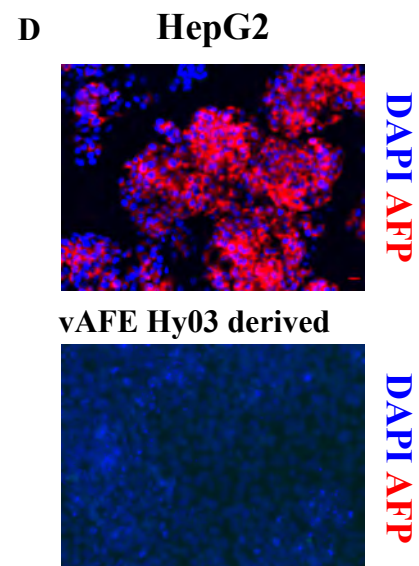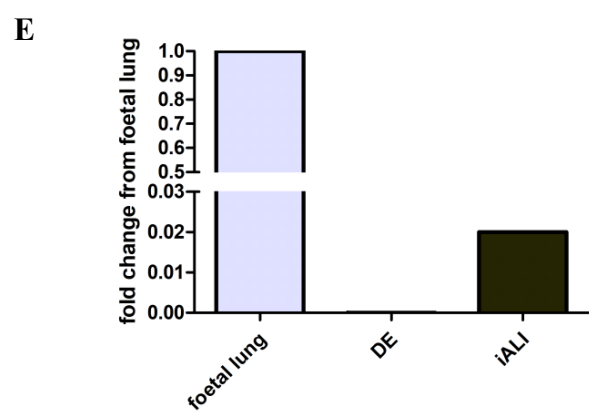

Supplement: Supplementary file 1 [file cells-11-02422-s001.zip › Bourdin De Vos Supplemental Figure S4.pdf]
